# Direct lentivirus injection for fast and efficient gene transfer into brown and beige adipose tissue

**DOI:** 10.14440/jbm.2016.123

**Published:** 2016-07-16

**Authors:** Aileen Balkow, Linda S. Hoffmann, Katarina Klepac, Anja Glöde, Thorsten Gnad, Katrin Zimmermann, Alexander Pfeifer

**Affiliations:** ^1^Institute of Pharmacology and Toxicology, University Hospital Bonn, University of Bonn, 53127 Bonn, Germany; ^2^Research Training Group 1873, University of Bonn, 53127 Bonn, Germany; ^3^BIGS DrugS International Graduate School, University of Bonn, 53127 Bonn, Germany; ^4^PharmaCenter, University of Bonn, 53127 Bonn, Germany

**Keywords:** beige adipose tissue, brown adipose tissue, gene transfer, lentivirus

## Abstract

Brown adipose tissue is a special type of fat contributing to energy expenditure in human newborns and adults. Moreover, subcutaneous white adipose tissue has a high capacity to adapt an energy-consuming, brown-like/beige phenotype. Here, we developed an easy to handle and fast to accomplish method to efficiently transfer genes into brown and beige fat pads *in vivo*. Lentiviral vectors are directly injected into the target fat pad of anesthetized mice through a small incision using a modified, small needle connected to a microsyringe, which is well suited for infiltration of adipose tissues. Expression of the target gene can be detected in brown/beige fat one week after injection. The method can be applied within minutes to efficiently deliver transgenes into subcutaneous adipose tissues. Thus, this protocol allows for studying genes of interest in a timely manner in murine brown/beige fat and could potentially lead to new gene therapies for obesity.

## BACKGROUND

There is a high medical need to develop new strategies to treat overweight and obesity as these conditions have reached pandemic dimensions. Obesity and its comorbidities, such as type 2 diabetes, cardiovascular disease and certain kinds of cancer, are a major threat to global health [1, 2]. Brown adipocytes are promising targets as they consume energy and could be used to increase energy expenditure and facilitate weight loss and thereby counteract obesity.

Brown adipocytes are rich in mitochondria and express specifically the uncoupling protein 1 (UCP1). UCP1 is activated by free fatty acids and uncouples the proton gradient within the mitochondria leading to generation of heat instead of ATP. Cold exposure leads to sympathetic activation of lipolysis and consequent activation of UCP1 by the released free fatty acids [3]. Newborn humans and small mammals possess brown adipose tissue (BAT) that produces heat to maintain body temperature [4]. Importantly, metabolically active BAT can also be found in human adults [5, 6, 7] and activation and/or recruitment of human BAT can increase whole body metabolism [8, 9].

Apart from interscapular BAT, inducible brown adipocytes, so called beige or brite (brown-in-white) cells, have been found disseminated in white adipose tissue (WAT). Beige cells also contain a high number of UCP1-expressing mitochondria and consume energy similar to “classical” brown adipocytes [10]. The number of beige adipocytes can be increased by different stimuli like cold exposure or by certain drugs [11]. Among WAT depots the subcutaneous, inguinal WAT (WATi) has a high susceptibility to browning in mice and men [12, 13, 14]. Even though the knowledge about brown and beige adipocytes has increased significantly during the last years there are still many open questions.

Lentiviruses are enveloped, single stranded RNA viruses that belong to the family of *Retroviridae*. An important difference between lentiviruses and simple retroviruses is that lentiviruses have the unique ability to infect non-dividing, terminally differentiated eukaryotic cells. Therefore, they are widely used as an efficient method for gene transfer [15].

Here we describe a protocol of direct lentivirus injection into subcutaneous brown and beige adipose depots for efficient gene transfer into brown/beige adipocytes. The role of target genes can thereby be studied in brown and beige fat *in vivo* as well as in whole body metabolism without the need to establish transgenic mouse lines. Furthermore, this method could also serve as a potential gene therapy approach which specifically targets subcutaneous beige or brown fat depots.

### Development of the protocol

Lentiviruses efficiently transfer genes into white and brown adipocytes *in vitro* [16, 17] and have been shown to transduce WAT [18, 19]. We have developed and optimized the protocol for lentivirus injection into BAT and to induce beige cells in WATi. Importantly, the custom adapted injection device allows for the injection of small volumes through a very small needle to avoid significant tissue damage. The set-up provides clear visual control during injection without contamination of the glass syringe by virus.

### Experimental design

The use of lentivirus allows employing a large variety of constructs. Lentivirus can either be used to overexpress genes or to knockdown genes using shRNA or miRNA [20]. For every experiment it is important to use respective controls. The buffers used for dissolving the viral particles, *e.g.*, phosphate buffered saline (PBS) or Hank’s balanced salt solution (HBSS), can be used for mock injections. Moreover, either “empty” viruses without transgene cassette or viral vectors carrying a reporter gene like green fluorescent protein (GFP) can be injected as controls. Especially for shRNAs or miRNAs a scrambled control vector should be used. It is possible to have intra-individual, internal controls within one mouse by injecting the gene of interest into only one fat lobe and the respective control into the other. This is useful if overexpression or knockdown efficiency within one mouse is investigated but should not be applied if whole body metabolism is studied.

Mice that have been injected with lentivirus can be included into long term studies, because the lentivector integrates into the host genome. Expression of the transgene is stable for at least 6 weeks [19].

### Comparison with other methods

Until now, fat tissue specific overexpression or knockdown of target genes was mostly accomplished by generating specific transgenic or knockout mouse models by subzonal injections of lentivirus into fertilized oocytes or using the Cre-lox recombination system, respectively [21]. Even though these methods are proficient in transferring or deleting the gene of interest into specific tissues by tissue-specific promoters, they are also very time consuming and expensive. Furthermore, a large number of mice are needed for breeding to establish a specific mouse line.

Direct lentiviral injection into brown/beige fat, does not involve breeding or genotyping of mouse lines and hence dramatically reduces the time required to express a gene of interest in fat pads as well as number of animals and costs. Another advantage of the method described here is the gene transfer into specific fat pads (*e.g.*, only WATi, but not BAT). So far this cannot be accomplished with transgenesis as there are no promoters available which specifically target only one of these fat pads, *e.g.*, the UCP1 promoter is active in both brown and beige cells [22].

The described set-up/method can also be used to deliver genes of interest into existing transgenic animals as well as for delivery of other viral vectors [23], like vectors derived from adenovirus or adeno-associated virus (AAV). AAV-derived particles are considered biohazard level 1; however, a major disadvantage is the small packaging capacity of classical AAV vectors [24, 25]. An important feature of lentiviral vectors is their integration into the host genome ensuring long-term expression. Moreover, one-and-the-same vector can be used to generate transgenic animals by subzonal injection (lentiviral transgenesis) [20].

Using the method described here, it is possible to generate genetically modified mice within minutes.

### Limitations

We recommend using mice of at least 4 weeks of age for injections into BAT and WATi. The presented method is primarily designed for subcutaneous fat tissues, which are easily accessible via a small skin incision. In principal, injections into visceral fat are possible, but injection into the epididymal adipose tissue would require opening the abdominal cavity. In the adipose tissue, not only adipocytes will be transduced with this method but all present cells (*e.g.*,preadipocytes, fibroblasts, immune cells, endothelial cells). Adipocyte-specific expression can be achieved with specific promoters to control expression of the target gene, *e.g.*, the fatty acid-binding protein 4 (aP2) promoter for all fat cells or the UCP1 promoter for only brown and beige fat cells [22]. To achieve cell-specific transduction, one could design viruses that have an adipocyte-specific fusogen that delivers the virus to designated cell types only [26]. ASC-1, PAT2, and P2RX5 could serve as specific surface markers for white, beige and brown adipocytes [27].

## MATERIALS

### General considerations on safety issues

All experiments have to be performed in accordance to relevant guidelines and protocols and have to be approved by local authorities including ethics/animal health committees. The study described herein has been approved by the Landesamt für Natur, Umwelt und Verbraucherschutz, NRW, Germany. Working with lentiviruses is restricted to biosafety level 2 (BSL-2) facilities. Appropriate personal protection equipment such as gloves and lab coat are required when working with these vectors. The paper “State-of-the-Art Lentiviral Vectors for Research Use: Risk Assessment and Biosafety Recommendations” by Katia Pauwels *et al.* (2009) [28] provides a good overview about risk assessment and biosafety recommendations when working with lentiviral vectors. Mice injected with replication-deficient virus are usually classified as BSL-1 organisms and can therefore be handled as any other BSL-1 laboratory mouse. However, as legislation might differ between countries, it is essential to review the local guidelines and precautions for handling viruses in BSL-2 facilities as well as lentiviral-injected mice before starting with the procedure.

### Animals

We have successfully overexpressed transgenes with direct lentivirus injection into brown (interscapular BAT) and beige (WATi) adipose tissue in C57Bl/6 mice at 4 weeks of age or older. Other mouse strains might be as suitable for the method as C57Bl/6 mice, but adjustment of the protocol might be necessary. For example when using ob/ob mice, it is necessary to adjust the number of viral particles depending on the increase in adipose tissue mass compared to wild-type controls. We recommend testing the procedure with Trypan blue solution or with a reporter construct (*e.g.*, GFP) before starting with the actual experiments.

### Reagents

• Ethanol 70%
• Isoflurane (Abbott), 1–2.5% (w/v) in oxygen. **CAUTION**: Isoflurane is harmful if inhaled, swallowed or upon skin contact. Wear appropriate personal protection equipment and use devices with carbon filters to minimize exposure to isoflurane.
• Trypan blue solution, 0.4%, **CAUTION**: Trypan blue might be hazardous to your health, wear appropriate personal protection equipment such as gloves when working with trypan blue.
• Packaging plasmids pMDLg/pRRE, RSV-rev and pMD.G for lentivirus production
• High titer lentivirus encoding the desired gene or reporter. The virus is produced according to established protocols [21]. The lentiviral vector was obtained by cloning GFP into the Bam HI and Sal I sites of the vector p156rrlsinPPT, which carries a ubiquitous CMV promoter. **CAUTION:** Adhere to biosafety regulations and only use the virus within a BSL-2 facilities. The virus can only be handled within a safety cabinet. Wear appropriate personal protection equipment such as gloves and lab coat.
• HBSS (Hank’s Balanced Salt Solution, Life technologies)
• Carprofen (Rimadyl, Pfizer)

### Reagent setup

*Virus solution*. High titer lentivirus should be prepared according to established protocols [21, 29]. In short, HEK 293T cells (ATCC) are seeded on poly-L-lysine-coated 150-mm2 dishes and co-transfected with the lentiviral vector plasmid as well as the packaging plasmids pMDLg/pRRE, RSV-rev and pMD.G. After transfection, cells are incubated at 37°C and 3% CO_2_ overnight. The transfection medium is then exchanged and cells are further incubated at 37°C and 10% CO_2_. The secreted virus is harvested after another 24 h and 48 h by collecting the supernatant of the cells. This supernatant is centrifuged by an ultracentrifuge (Beckman Coulter) with SW32Ti rotor at 61,700 g at 17°C for 2 h to pellet the secreted virus. The virus pellet is resuspended in HBSS. Combined virus suspensions after 24 h and 48 h are concentrated by centrifugation over a 20% (w/v) sucrose cushion in a SW55Ti rotor (Beckman Coulter) at 53,500 g at 17°C for 1.5 h and again solved in HBSS. The physical titer of the lentivirus can be assessed by colorimetric reverse transcriptase (RT) assay (Roche). Dilute lentivirus in HBSS, to achieve desired volume for injection (*i.e.* 20–30 µl for each fat lobe). In this protocol 1000 ng/25 µl (BAT) and 1000 ng/30 µl (WATi) of lentivirus were injected into each fat lobe.

### Equipment

• Surgical swabs (Paul Hartmann)
• Animal hair clipper (AESCULAP GT420)
• Isoflurane anesthesia system (Vapor, Dräger)
• Nanopass33 (33G needle for pen injectors, Terumo Corp.)
• Fine-Bore Polyethylene (Polythene) Tubing (ID 0.28 mm, OD 0.61 mm, Smiths Medical)
• Microsyringe (Hamilton)
• Tungsten carbide scissors (Fine Science Tools)
• Surgical scissors (Fine Science Tools)
• Curved forceps (Fine Science Tools)
• Straight forceps (Fine Science Tools)
• Michel suture clips (Fine Science Tools)
• Michel clip applicator (Fine Science Tools)
• Heating pad

### Equipment setup

**Figure 1:**
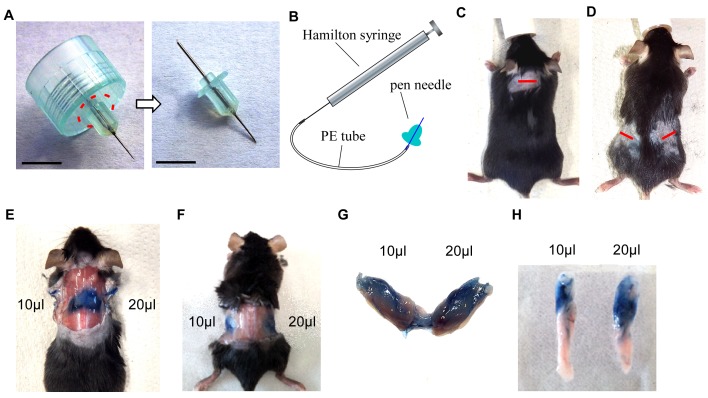
**Set up of injection device and establishment of protocol**. **A**. Original pen needle (dashed line indicates the cutting area) (left) and needle after cutting off plastic rim (right). Scale bar = 0.5 cm. **B**. Illustration showing setup of injection device. **C** and **D**. Pictures of anesthetized and shaved mouse, incision sites are marked by a red line in the interscapular region (C) or at the flanks, proximal of hips (D). **E**. *Situs* after injection of 10 μl and 20 μl Trypan blue solution in BAT. **F**. *Situs* after injection of 10 μl and 20 μl Trypan blue solution in the upper region of WATi. **G**. Dissected BAT from (E). **H**. Dissected WATi from (F).

*Injection device.* Cut off outer plastic rim around the pen-needle (**Fig. 1A**) and connect it to the polyethylene (PE) tubing (20–30 cm in length). Be careful not to puncture the tubing with the pen needle which is sharp at both sides. Connect a Hamilton microsyringe to the other side of the tubing (**Fig. 1B**). To test whether all connections are tight and correctly sealed, flush the injection device with PBS. The virus solution can now be drawn up with the microsyringe into the plastic tubing and will be visible within the tubing. The injection device is now ready to use.

**CAUTION**: Adhere to biosafety regulations in terms of handling and disposal of virus and virus containing material.

## PROCEDURE

### *In vitro* testing of lentiviral vectors

We recommend testing your lentiviral construct *in vitro* before injecting it into mice. Such a test could be performed as follows:

1. Seed preadipocytes and let attach.
2. Transduce with different amounts of virus (*e.g.*, 50 ng and 200 ng per 6-well).
3. Differentiate the cells according to standard differentiation protocol [19, 30].
4. Expression of the transgene can be quantified using qRT-PCR for mRNA expression, Western blotting for protein expression (**Fig. S1A**-**S1B**) or microscopic analysis at different time points during differentiation if using a reporter gene (**Fig. S1C**-**S1D**).

### *In vivo* injection of lentiviral vectors

5. Inject mice with analgesic (Carprofen 5 mg/kg body weight) 30 min before starting the procedure.
6. Under the safety cabinet draw up the desired amount of virus. We recommend using a volume of 20–30 µl in each fat lobe of BAT and WATi.
7. Anaesthetize mice with isoflurane: Anesthesia is induced with 2.5% (w/v) isoflurane and maintained with 1% (w/v) isoflurane in O_2_. **CAUTION**: Test if anesthesia is deep enough by pinching the skin between the toes. No withdrawal should be observed.
8. Shave the small area in the interscapular region for injections into BAT (**Fig. 1C**) and at the flanks, proximal of hip joints for injections into WATi/beige fat with hair clipper (**Fig. 1D**). Clean the skin with ethanol.
9. Make a 0.513970591400.8 cm incision in the skin using surgical scissors (see red lines in **Fig. 1C** and **1D**).
10. Hold skin open with tweezers to expose fat pad. Visualize BAT with surrounding/adjacent white fat pads (**Fig. 1E**) or the dorsal tip of the WATi pad (**Fig. 1F**). For injections into WATi use tweezers to shift the fat pad upwards through the incision and expose the pad without completely removing it from its native position.
11. Take the injection device and carefully insert the pen needle into the fat pad. Only when the needle is inserted deep enough start to inject lentiviral vectors into multiple (5139705914010) distinct spots in the fat pad using the fine needle (total 20139705914030 µl per lobe).
12. After the virus solution is injected completely, carefully take out the needle and dispose the needle together with the tubing according to local regulations.
13. Close the incision with Michel suture clips.
14. Post surgery, monitor health status of the mice daily and treat the mice with analgesic (Carprofen 5 mg/kg body weight) for two days or longer if you have hints that the mouse is still in pain (not normal behavior, *e.g.*, less exploring and rearing; outer appearance, *e.g.*, shaggy fur, blurry eyes, inflammation of the wound). Recovery from the small surgery is usually very quick and the suture clips can be removed or are lost generally after 7139705914014 days. **CAUTION**: always refer to approval by local ethics/animal health committees.
15. Analysis: The injected mice can be retained for any desired time period from days up to several weeks and can even be used for further *in vivo* experiments after the appropriate recovery time (*e.g.*, metabolic measurements, special dietaries, glucose tolerance tests).

### Timing

Steps 5-6, preparation and pre-treatment of mice: 30 min

Steps 7-13: 10-15 min

Step 14: 2 days or longer

Step 15: Any desired time point

**Figure 2:**
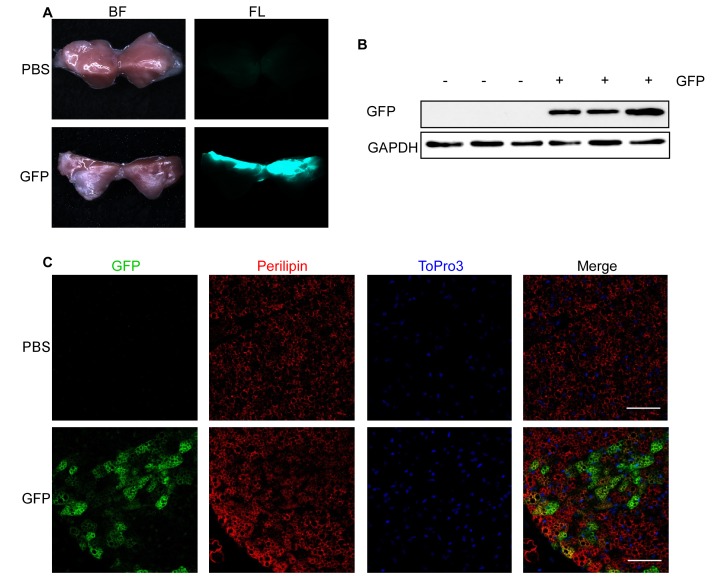
**GFP fluorescence in interscapular BAT after lentivirus injection**. **A**-**C**. 1000 ng RT of lentivirus carrying GFP under control of a CMV promoter (GFP) in 25 μl HBSS or 25 μl PBS were injected into each interscapular BAT lobe of 4-week-old male mice and analyzed after 1 week. **A**. Bright field (BF, left) and fluorescent images (FL, right) of PBS (upper panel) and GFP (lower panel) injected BAT. **B**. GFP expression assessed by Western blotting. Respective blots of GFP and the loading control GAPDH are shown. **C**. GFP expression assessed by immunohistological staining. PFA-fixed BAT sections were triple stained with antibodies directed against GFP (green) and Perilipin (red) as well as with ToPro3 (blue) to stain the nuclei. Scale bar = 50 µm.

## ANTICIPATED RESULTS

### *In vitro* testing of lentiviral vectors

To test that your lentiviral vector is working in principle, it is recommended to test it *in vitro* before injecting it into your mice. We transduced brown (BA) and inguinal white adipocytes (WAi) with two different amounts of lentivirus carrying GFP under the control of a cytomegalovirus (CMV) promoter (*i.e.*, 50 ng and 200 ng per 150,000 seeded cells) and differentiated the cells to mature adipocytes according to standard protocol. Proteins from terminally differentiated cells were isolated and Western blotting was performed to analyze GFP expression, which was only present in transduced BA and WAi but not in untransduced control (ctrl) cells (**Fig. S1A**-**S1B**). During differentiation GFP expression was confirmed by means of fluorescent microscopy, showing expression of GFP in preadipocytes (d-2) as well as in mature BA and WAi (d7) (**Fig. S1C**-**S1D**).

### Establishment of protocol using Trypan blue solution

Before injecting precious lentivirus solutions, we recommend to get used to the protocol with the use of Trypan blue solution. After following the protocol as outlined in the PROCEDURE section the adipose tissue pads should appear in a blue color as shown in **Figure 1E**-**1H**.

### GFP expression in BAT and WATi

**Figure 3:**
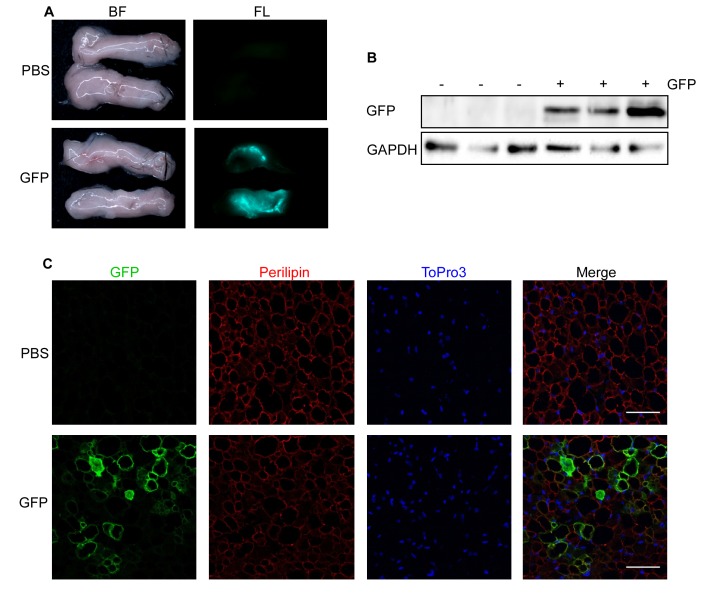
**Expression of GFP in inguinal WAT (WATi) after lentivirus injection.** **A**-**C**. 1000 ng RT of lentivirus carrying GFP under control of a CMV promoter (GFP) in 30 μl HBSS or 30 μl PBS were injected into each WATi pad of 10-week-old male mice and analyzed after 1 week. **A**. Bright field (BF, left) and fluorescent images (FL, right) of PBS (upper panel) and GFP (lower panel) injected WATi. **B**. GFP expression assessed by Western blotting. Respective blots of GFP and the loading control GAPDH are shown. C. GFP expression assessed by immunohistological staining. PFA-fixed WATi sections were triple stained with antibodies directed against GFP (green) and Perilipin (red) as well as with ToPro3 (blue) to stain the nuclei. Scale bar = 50 µm.

**Figure 4:**
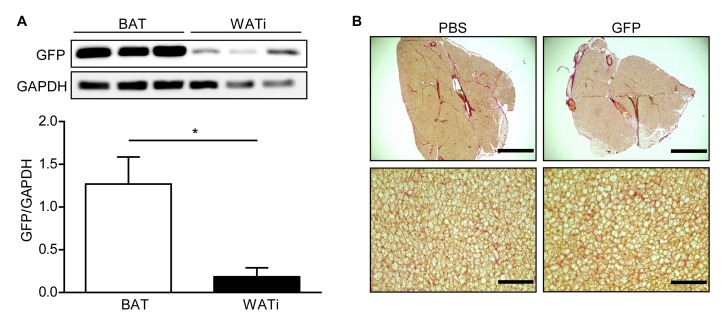
**Comparison of GFP expression in BAT and WATi and Sirius Red staining of BAT**. **A**. GFP expression in BAT and WATi after injection of 1000 ng RT of lentivirus carrying GFP into each fat lobe assessed by Western blotting (top) and quantitative analysis (bottom). * *P* < 0.05 (n = 3). **B**. Representative BAT sections of PBS- and GFP-injected mice, stained with Sirius Red for collagen (I and III) fibres. Scale bar top = 1 mm; scale bar bottom = 100 µm.

One week after injection of 1000 ng lentivirus carrying GFP under the control of a cytomegalovirus (CMV) promoter per BAT lobe, fluorescence was clearly visible in the dissected fat pads (**Fig. 2A**). The results were validated using Western blotting (**Fig. 2B**), which showed expression of GFP in lentivirus-injected mice but not in mice injected with PBS. Furthermore, we performed immunofluorescent staining of GFP, Perilipin (adipocyte marker) and ToPro3 (nucleus staining) in PFA-fixed sections of lentivirus-injected BAT pads to analyze brown adipocyte specific GFP expression (**Fig. 2C**). Similar results were obtained after injection of WATi (**Fig. 3A**-**3C**). Even though the same amount of lentivirus (*i.e.*, 1000 ng/fat lobe) was injected into BAT and WATi, expression levels of GFP were significantly lower in WATi in comparison to BAT (**Fig. 4A**). This is probably due to the larger overall size of WATi, which reduces the number of viral particles per gram of tissue. We recommend using a higher amount of lentivirus for injections into WATi than into BAT. Sirius Red staining of lentivirus-injected BAT pads detected no damage in the injected tissue due to the small injection needle used (**Fig. 4B**).

In conclusion, we present a fast and easy applicable method to efficiently transfer genes into murine brown and beige adipocytes using lentiviral vectors.

## TROUBLESHOOTING

Potential problems and their causes and solutions are listed in **Table 1**.

**Table 1:** Troubleshooting.

Step	Problems	Causes	Suggestions
6	Impossible to draw up virus	• Virus solution is too viscous	• Dilute in larger volume of virus solvent
		• PE tubing is damaged	• Use a new piece of tubing and be very careful when connecting it to the pen needle
		• Pen needle is damaged or clogged	• Use a new pen needle
7	Anaesthesia does not work	• Isoflurane dose is too low	• Carefully increase dose of isoflurane until mouse loses consciousness and does not react to pinch test anymore. Check mouse constantly for signs of awakening.
		• Isoflurane anesthesia system is not set up properly	• Check all connections
14	Incision becomes inflamed	• Surgical instruments were not properly cleaned	• Sterilize and autoclave all used surgical instruments properly
		• Hair was not properly removed and entered the wound	• Remove fur in a larger area around the incision. Use ethanol to clean sticking hair
		• Transferred gene induces inflammation
15	No or too low expression of transferred gene	• Fat pads were not injected properly	• Practice the method using Trypan blue solution (see **Fig. 1E**-**1H**) to make sure the correct spots are injected. Be careful not to put the needle too deep or too shallow. Try to inject in as many different spots as possible to ensure expression in the whole tissue.
		• Low expression efficiency of lentiviral vector	• Test efficiency of lentiviral vector *in vitro,*if possible try to increase amount of injected lentivirus
		• Analysis is done too early	• We recommend waiting at least 48–72 h post injection before starting with analysis to ensure proper transduction of the tissues and expression of the transferred gene.

## Abbreviations used

BAT: brown adipose tissue
UCP1: uncoupling protein 1
WAT: white adipose tissue
WATi: inguinal white adipose tissue
PE: polyethylene
GFP: green fluorescent protein
CMV: cytomegalovirus
aP2: fatty acid-binding protein 4
AAV: adeno-associated virus

## Supplementary information

**Figure S1**. Example for testing of lentiviral constructs in vitro in brown and white adipocytes
